# *Notes from the Field*: Increases in Firearm Homicide and Suicide Rates — United States, 2020–2021

**DOI:** 10.15585/mmwr.mm7140a4

**Published:** 2022-10-07

**Authors:** Thomas R. Simon, Scott R. Kegler, Marissa L. Zwald, May S. Chen, James A. Mercy, Christopher M. Jones, Melissa C. Mercado-Crespo, Janet M. Blair, Deborah M. Stone

**Affiliations:** ^1^Division of Violence Prevention, National Center for Injury Prevention and Control, CDC; ^2^Division of Injury Prevention, National Center for Injury Prevention and Control, CDC; ^3^Office of the Director, National Center for Injury Prevention and Control, CDC.

The firearm homicide rate in the United States increased nearly 35% from 2019 to 2020, coinciding with the emergence of the COVID-19 pandemic (*1*). This increase affected all ages and most population groups, but not equally: existing disparities, including racial and ethnic disparities, widened. The firearm suicide rate was higher than the firearm homicide rate in 2020 and remained consistent with recent years overall; however, increases were observed in some groups (*1*). To assess potential increases from 2020 to 2021, final 2020 and provisional 2021, National Vital Statistics System mortality data and U.S. Census Bureau population estimates were used to examine all-cause homicide and suicide rates; firearm homicide and suicide rates overall and by sex, age,* race and ethnicity; and the percentage of homicides and suicides from firearm injuries.^†^ This activity was reviewed by CDC and was conducted consistent with applicable federal law and CDC policy.^§^

An estimated 20,966 firearm homicides and 26,320 firearm suicides occurred in the United States during 2021 (Table). From 2020 to 2021, the percentage of homicides and suicides attributed to firearm injuries increased from 79% to 81% and from 53% to 55%, respectively, resulting in the highest percentage for homicide in more than 50 years and the highest percentage for suicide since 2001.

**TABLE:** All-cause and firearm-related homicides and suicides — United States, 2020–2021

Event/Sex	2020		2021*		% Change in rate from 2020 to 2021
	No. (rate^†,§^)	% By firearm^¶,^**	No. (rate^†,§^)	% By firearm^¶,^**	
**All-cause homicides**					
**Total**	**24,574 (7.69)**	**78.9**	**25,987 (8.14)**	**80.7**	**5.9**
**Firearm homicides**					
**Total**	**19,383 (6.12)**	**—**	**20,966 (6.63)**	**—**	**8.3**
Male	16,427 (10.29)	—	17,604 (11.04)	—	7.3
Female	2,956 (1.85)	—	3,362 (2.11)	—	14.0
**All-cause suicides**					
**Total**	**45,957 (15.63)**	**52.9**	**48,023 (16.31)**	**54.8**	**4.3**
**Firearm suicides**					
**Total**	**24,292 (8.07)**	**—**	**26,320 (8.75)**	**—**	**8.3**
Male	21,180 (14.50)	—	22,930 (15.65)	—	8.0
Female	3,112 (2.08)	—	3,390 (2.27)	—	8.9

**Sources:** CDC WONDER; CDC Web-based Injury Statistics Query and Reporting System (WISQARS); U.S. Census Bureau.

* Data for 2021 are provisional and as reported through August 7, 2022.

^†^ Homicide rates are per 100,000 persons. Rates include all decedents with documented age. Rates are age-adjusted to the year 2000 U.S. standard population.

^§^ Suicide rates are per 100,000 persons aged ≥10 years. Rates are age-adjusted to the year 2000 U.S. standard population. Suicide statistics exclude data for persons aged <10 years because intent for self-harm can be difficult to ascertain in young children.

^¶^ Homicide percentages are based on all homicides with or without documented decedent age.

** Suicide percentages are based on all suicides with documented decedent age ≥10 years.

The firearm homicide rate in 2021 was 8.3% higher than it was in 2020 (Table); increases occurred among both males and females. The highest rates were generally among persons aged 25–44 years, with increases occurring in each racial and ethnic population in that age group (Supplementary Table, https://stacks.cdc.gov/view/cdc/121555). Non-Hispanic Black or African American persons continued to experience the highest firearm homicide rates in every age group.

The firearm suicide rate among persons aged ≥10 years also increased 8.3% from 2020 to 2021 (Table), with increases among males and females, and most age by race and ethnicity groups (Supplementary Table, https://stacks.cdc.gov/view/cdc/121555). The highest firearm suicide rates for persons aged <45 years were among non-Hispanic American Indian or Alaska Native (AI/AN) persons, and the highest rates for those aged ≥45 years were among non-Hispanic White persons.

The overall U.S. firearm homicide and firearm suicide rates in 2021 were the highest documented since 1993 and 1990, respectively. Some racial and ethnic groups experienced substantially higher rates in 2021, and among some groups, disparities continued to widen. This analysis cannot explain the reasons for the increases; however, multiple social and structural conditions are associated with risk for homicide and suicide. Systemic inequities (e.g., in economic, educational, housing, and employment opportunities) and structural racism have contributed to disparities in outcomes, and the COVID-19 pandemic could have worsened these conditions, especially in some racial and ethnic communities (*1*,*2*).

The findings in this report are subject to at least three limitations. First, the 2021 data in this report are provisional and might change when final data are available; however, reported rates are unlikely to shift downward. Second, rates for some population groups could not be reported because of small counts. Finally, some racial and ethnic groups, particularly AI/AN persons, might be undercounted because of misclassification (*3*).

Increases since 2020 and record high rates of firearm homicide and suicide in 2021 underscore the urgent need for prevention efforts. Public health can facilitate collaboration across sectors, including health, law enforcement, education, social services, and community organizations, to implement a coordinated and comprehensive approach based on the best available evidence. To help communities make use of the best available evidence for violence prevention, CDC has released Technical Packages for Violence Prevention.^¶^ Prevention efforts can include street outreach and hospital-based interventions, efforts to enhance secure firearm storage and reduce access to firearms among those at risk for harming themselves or others, changes to the physical environment (e.g., remediating vacant lots to enhance safe spaces), programs that enhance positive social connections or teach coping and problem-solving skills, therapeutic interventions (e.g., crisis intervention and treatment to address previous trauma), and policies (e.g., housing and economic) that address underlying risks and inequities.

## Footnotes

* Children aged <10 years were excluded from analysis of suicides because self-harm intent can be difficult to ascertain in young children.

† CDC, National Center for Health Statistics, National Vital Statistics System, Provisional Mortality on CDC WONDER Online Database, https://wonder.cdc.gov (Accessed August 11, 2022); CDC, National Center for Injury Prevention and Control, Web-based Injury Statistics Query and Reporting System (WISQARS), https://www.cdc.gov/injury/wisqars/index.html (Accessed August 11, 2022); U.S. Census Bureau, Population Division, Annual Estimates of the Resident Population by Single Year of Age and Sex for the United States: April 1, 2020 to July 1, 2021 (NC-EST2021-AGESEX-RES), https://www.census.gov (Accessed July 9, 2022); U.S. Census Bureau, Population Division, Annual Estimates of the Resident Population by Sex, Age, Race, and Hispanic Origin for the United States: April 1, 2020 to July 1, 2021 (NC-EST2021-ASR6H), https://www.census.gov (Accessed August 7, 2022).

§ 45 C.F.R. part 46, 21 C.F.R. part 56; 42 U.S.C. Sect. 241(d); 5 U.S.C. Sect. 552a; 44 U.S.C. Sect. 3501 et seq.

¶
https://www.cdc.gov/violenceprevention/communicationresources/pub/technical-packages.html
